# SARM1: a key multifaceted component in immunoregulation, inflammation and neurodegeneration

**DOI:** 10.3389/fimmu.2025.1521364

**Published:** 2025-05-13

**Authors:** Samuel dos Santos Oliveira, João Vinícius Honório da Silva, Raquel de Souza Vieira, Luís Felipe Serra Moreira, Pedro Henrique Araújo Bandeira, Beatriz Leocata Ramos, Marco Antônio Ataíde Silva, Niels Olsen Saraiva Câmara

**Affiliations:** ^1^ Department of Biochemistry and Immunology, Ribeirão Preto Medical School – FMRP of the University of São Paulo – USP, Ribeirão Preto, Brazil; ^2^ Department of Immunology, Institute of Biomedical Science – ICB of the University of São Paulo – USP, São Paulo, Brazil

**Keywords:** innate immunity, immunometabolism, cell death, immunoregulation, neurodegenerative disease

## Abstract

The downstream signaling pathways of TLR activation involve a family of adaptor proteins, including MYD88, TIRAP, TRIF, TRAM, and SARM1. The first four proteins stimulate inflammatory and antiviral responses, playing crucial roles in innate immunity against various pathogens. In contrast, SARM1 promotes immunity to microorganisms in invertebrate animals independently of TLRs, and negatively regulates inflammatory responses in metazoan organisms. SARM1 inhibits TRIF, reduces the activation of various inflammasomes, and induces mitochondrial damage and cell death to eliminate hyperactivated cells. This regulation is essential to ensure timely control of immune responses and to prevent excessive inflammation. Recently, it was discovered that SARM1 can hydrolyze NAD, a critical component of cellular metabolism. The reduction of NAD levels by SARM1 is linked to the progression of Wallerian degeneration following neuronal injury and may also play a role in the immunoregulation of lymphoid and myeloid cells. Since SARM1 can be pharmacologically modulated, it presents promising opportunities for developing treatments for inflammatory and neurodegenerative diseases.

## 1 Introduction

The immune system in mammals is a highly sophisticated recognition network equipped with an extensive array of cellular and molecular machinery, primarily tasked with maintaining the host organism’s homeostasis (1). In humans, this monitoring becomes particularly challenging due to constant environmental changes, such as fluctuations in temperature, diet, circadian rhythm, microbiota, and the emergence of infections (2).

The immune system plays a pivotal role in safeguarding the organism by recognizing stress-causing agents, whether infectious or otherwise, and restoring equilibrium (1). However, dysregulation within this intricate system can compromise its ability to neutralize stressors or properly quell inflammatory responses once the threat has been addressed (3, 4). Unchecked inflammation may lead to substantial tissue damage and the onset of autoimmune or autoinflammatory disorders, resulting in significant morbidity and mortality worldwide (5).

Pathogen elimination is one of the most widely discussed functions of the immune system (6), with Toll- (TLRs), Nod-Like Receptors (NLR) and Inflammasomes being key components due to their ability to recognize various classes of pathogens (7). Specifically, TLRs detect conserved molecules called pathogen-associated molecular patterns (PAMPs) and alert the host to the presence of potentially dangerous organisms (8). TLR engagement represents a pivotal step in innate immunity, capable of either promoting inflammation or initiating an antiviral state. The effector mechanism triggered depends on the type of PAMP and the respective TLR involved in its recognition. These receptors also play a crucial role in activating antigen-presenting cells (APCs), thereby enhancing their ability to activate T lymphocytes and instructing dendritic cells (DCs) on the appropriate differentiation profile for recognizing specific pathogens (9).

TLRs are anchored in both cytoplasmic and endosomal membranes, and upon engagement, they trigger a signaling cascade mediated by homotypic interactions between adaptor proteins containing Toll/IL-1R (TIR) domains present in the cytosol (10, 11). Although more than 7 proteins belonging to this family have been described, Myeloid Differentiation Factor 88 (MYD88), Toll/IL-1R domain-containing adaptor protein (TIRAP), Toll/IL-1R domain-containing adaptor inducing interferon-β (TRIF), TRIF-related adaptor molecule (TRAM), and Sterile Alpha and TIR Motif-containing protein 1 (SARM1) are the most widely recognized and are of significant importance in the induction and regulation of inflammation (6).

MYD88 participates in signal transduction originating from the activation of IL-1 receptor (IL-1R) and TLRs (12). When activated, TLRs undergo dimerization, and some of them (TLRs 7, 8, and 9) can directly recruit MYD88, which then recruits TRAF6 to promote inflammation or TRAF3 to induce the antiviral state. TLRs 1, 2, 4, and 6 utilize the adaptor protein TIRAP to facilitate MYD88 recruitment and subsequent IRAK attachment, which induces NF-KB activation (13–16). TLR3 signaling is associated with detecting viral infections by recognizing double-stranded RNA. Upon activation, they recruit TRIF, an adaptor capable of interacting with TRAF3 to induce an antiviral response through the activation of IRF3/7, but also capable of interacting with TRAF6 to activate NF-kB and promote inflammation (17). TRIF is also essential in signaling originating from TLR-4 internalized in endosomes, in this case, another adaptor, TRAM, facilitates TRIF recruitment, which interacts with TRAF6 to promote inflammation via NF-kB (18–21).

SARM1, in turn, diverges from what has been discussed regarding its relatives concerning its functionality in the immune response. In phylogenetically distant organisms, the presence of a functional gene homologous to human SARM1 confers antimicrobial activities, albeit independently of TLRs (6, 22–24). In vertebrate metazoans, SARM1 acts in different instances to regulate the immune response, potentially inhibiting TLR responses, NLRP3, and AIM2 inflammasome responses (22, 25), promoting the death of hyperactivated cells by disrupting mitochondrial homeostasis (26, 27). Some evidence suggests that SARM1 may also contribute to the contraction of the adaptive response after eliminating an infectious agent (28). In this review, we will address the different functions exerted by SARM1 in different cells and organisms. We will also discuss the therapeutic potential of modulating its expression and activation for the treatment of inflammatory and neurodegenerative diseases.

## 2 Regulation of TLR response by SARM1

SARM1 was initially described in 2001 as an evolutionarily conserved protein present in phylogenetically distant species such as *Homo sapiens*, *Mus musculus*, *Drosophila melanogaster*, and *Caenorhabditis elegans*. Its functionality was elucidated with the discovery of the SARM1 ortholog in fruit flies, GG7915, and its ability to affect the insect’s embryonic development, as animals deficient in this gene were embryonically inviable (23). Knowledge about the structure and functionality of SARM1 has been greatly expanded through studies with *C. elegans*. Structurally, Toll-interleukin 1 repeat (TIR-1), the SARM1 ortholog in *C. elegans*, also expresses the TIR, Armadillo repeat (ARM), and two Sterile alpha motif (SAM) domains. The SAM domains consist of 70 amino acids and function in the formation of homodimers (29). Besides being present in adaptor molecules like SARM1 itself, these domains have also been described in transcription factors (30), tyrosine kinases (31), MAP kinases, and serine/threonine kinases (32–35). The ARM domain is also present in a wide variety of proteins in animals and plants (36–38), while the TIR domain is somewhat more restricted. It is present in three types of proteins: transmembrane proteins with extracellular Ig domains, such as the IL-1 receptor (IL-1R); transmembrane proteins with extracellular leucine-rich repeat (LRR) domains, such as TLRs; and cytoplasmic proteins responsible for TLR and IL-1R signal transduction (24, 39). The TIR domain comprises 200 amino acids and is responsible for homotypic interactions between proteins by affinity (33).

Generally, proteins containing the TIR domain play a central role in innate immunity. SARM1 in *C. elegans* has two proteins with this domain: Toll-1, a single TLR expressed by the animal, and TIR-1, which is homologous to SARM1. At the moment, we know that TIR-1 is a TLR adapter; however, the importance of this cytoplasmic protein in pathogen response independently of Toll-1 has been demonstrated. In fungal infections of *C. elegans* by *Drechmeria coniospora*, TIR-1 induces the expression of NLP-29 and NLP-31, components with high microbicidal activity. In this context, inhibition of Tir-1 expression by RNA interference (RNAi) renders the animals extremely susceptible to infection, and the absence of *C. elegans’* sole TLR did not alter the worms’ susceptibility to the fungus (40, 41).

**Figure 1:**
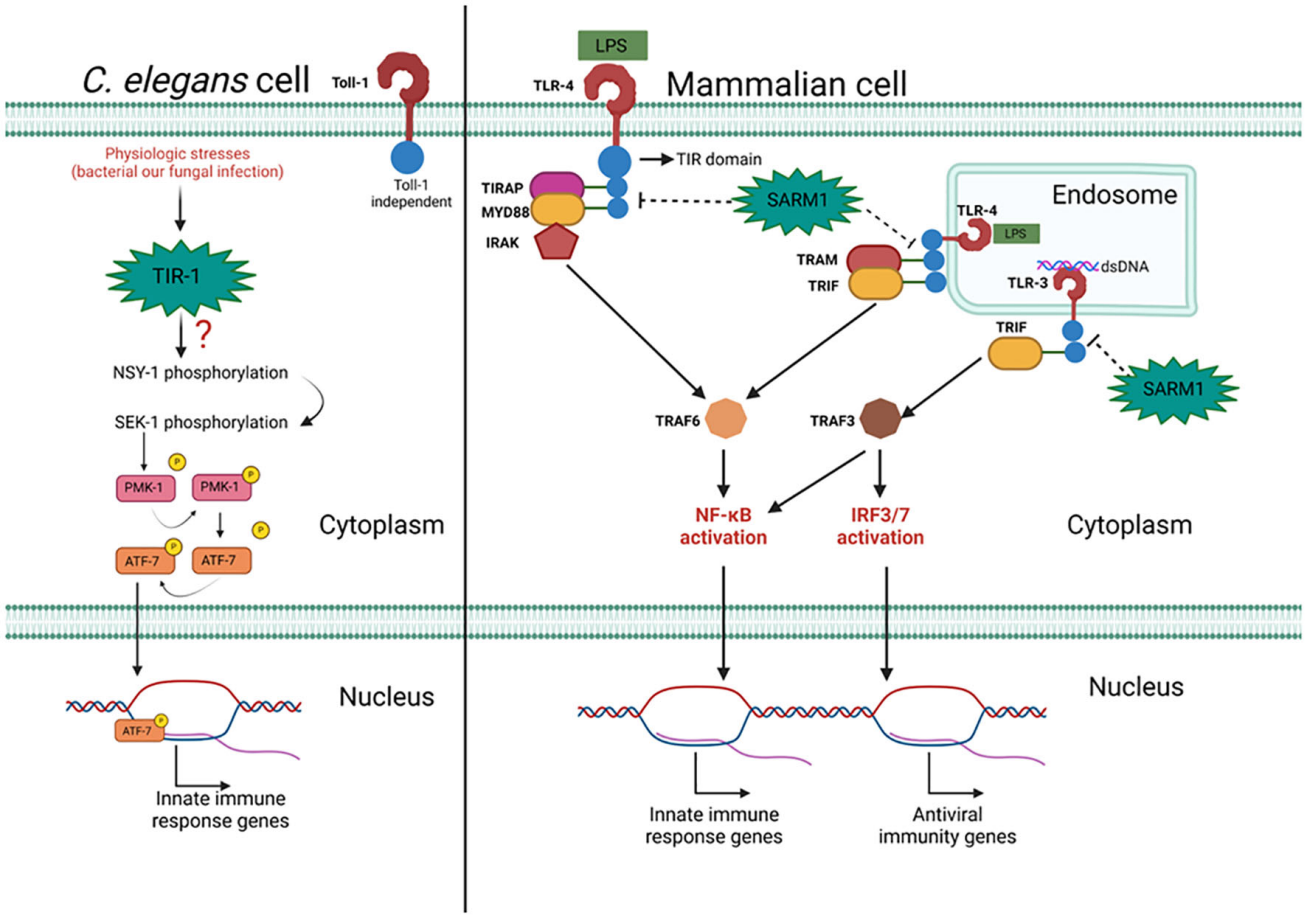
The action of mammalian SARM1 and its C. elegans ortholog TIR-1 in the immune response to pathogens. Pathogen presence triggers an inflammatory response mediated by TIR-1 independently of Toll-1. The mechanism by which TIR-1 is associated with sequential phosphorylation of NSY-1, SEK-1, and PMK-1 remains unknown. PMK-1 phosphorylates ATF-7, facilitating its translocation to promote the expression of inflammatory response genes. In mammalian cells, TLR activation promotes an inflammatory or antiviral response. Signal transduction from the plasma/endosomal membrane to the nucleus relies on adaptor proteins such as MYD88, TRIF, TRAM, and TIRAP. These adaptors interact via the TIR domain with TLRs and activate TRAF3 or TRAF6, important proteins for NF-kB and IRF3/7 activation. SARM1 interacts with TLR adaptor proteins through its TIR domain, inhibiting their recruitment to TLRs and blocking their signaling. Figure created with BioRender.com.

TIR-1 serves a crucial role in the antibacterial response, as demonstrated by experiments employing RNA interference (RNAi) to inhibit its expression, which heightened susceptibility to *Pseudomonas aeruginosa* infection in worms. This heightened susceptibility correlated with a significant decrease in levels of phosphorylated PMK-1, the biologically active form of PMK-1, without altering the total protein levels. This suggests that TIR-1’s action precedes the activation of PMK-1. PMK-1 is one of three enzymes belonging to the p38 family of Mitogen-Activated Protein Kinases (MAPK) in *C. elegans*, with the other two members, NSY-1 (MAPK3) and SEK-1 (MAPK2), also playing key roles in the worms’ immune response (42). Afterwards, a genetic screening study has identified mutations in these two MAP kinases being linked to the reduced worm survival against bacterial infection. Both NSY-1 and SEK-1 act downstream of PMK-1, as evidenced by RNAi silencing experiments that resulted in a marked reduction in PMK-1 phosphorylation (43) and increased susceptibility to *P. aeruginosa* infection (44). Conversely, overexpression of components within this pathway has been associated with a protective phenotype (45). Importantly, sequential phosphorylation of NSY-1, SEK-1, and PMK-1 leads to the phosphorylation of the transcription factor ATF-7, promoting the transcription of numerous genes associated with the innate immune response (46). This pathway also proves vital in the response to the Gram-positive bacterium *Enterococcus faecalis*, underscoring the central role of TIR-1 in antimicrobial immunity against a diverse array of pathogens (24) (see **Figure 1**).

SARM1 contributes to pathogen immunity in invertebrates in a TLR-independent manner and without inducing NF-kB and IRF3 activation (47, 48). Genes regulated by these transcription factors did not show increased expression after overexpressing human SARM1 in mammalian cells, indicating that its antimicrobial activity observed in some invertebrates stems from a mechanism very different from those observed for other members of the TIR domain-containing adaptor family (24, 29).

The expression of SARM1 is increased in mammalian cells following LPS stimulation, suggesting that SARM1 may play a role in regulating signal transduction between TLR4 and certain related transcription factors, such as NF-kB and IRF3. This phenomenon was confirmed by identifying a dose-dependent reduction in the expression of genes regulated by IRF3 after SARM1 overexpression. This effect is mediated by the homotypic interaction between the TIR domains of SARM1 and TRIF, potentially preventing TRIF from activating IRF3. Furthermore, NF-kB activation induced by TRIF is also dose-dependently diminished in cells with increased SARM1 expression. Thus, the initial functional activity of SARM1 in mammalian cells has been revealed, portraying it as an immunoregulatory component capable of competing with TRAM for interaction with TRIF, thereby aiding in the modulation of the innate immune response (29).

Further substantiating its immunoregulatory role, SARM1 was found to inhibit AP-1 activation by suppressing p38 MPK phosphorylation post TLR4 stimulation with LPS, independently of TRIF and MYD88. This type of regulation stands distinct from previous attributions to SARM1 (47). Notably, the homolog of SARM1 in *Larimichthys crocea*, Lc-SARM, has also been implicated in immunoregulation. Lc-SARM inhibits the activation of IRF3, IRF7, and NF-kB-dependent antiviral responses by interacting with TRIF (48) (see **Figure 1**). Most of the results obtained from assessing SARM1 function in the immune response in mammalian cells pointed to an immunoregulatory action mediated by direct interaction with TRIF. However, when using a SARM1 overexpression program in HEK293 cells, it was possible to observe that this adaptor can also interact with MYD88 through the glycine residue located in the BB loop of the TIR domain. This heterologous expression system of SARM1 promotes a reduction in the production of pro-inflammatory cytokines such as IL-8 and TNFα induced by LPS, a phenomenon that was reversed when cells were transduced with SARM1 containing the mutated BB loop (49).

In mice, SARM1 regulates the production of pro-inflammatory cytokines. SARM1 deficiency in macrophages exacerbated DSS-induced intestinal inflammation. Mechanistically, SARM1 promotes the recruitment of TRAF to MYD88 and negatively regulates the MYD88-dependent inflammatory response in the intestine (50).

## 3 SARM1 as a cellular steward and guardian of host homeostasis

In mammalian cells, SARM1 operates on multiple fronts to attenuate the immune response (25, 48, 49).This is a physiologically crucial mechanism, as inflammatory responses must be finely tuned. Excessive responses to a stimulus or difficulties in terminating the response after the elimination of the stressor (infection, tissue injury, and others) can inflict serious harm on the organism (1).

**Figure 2:**
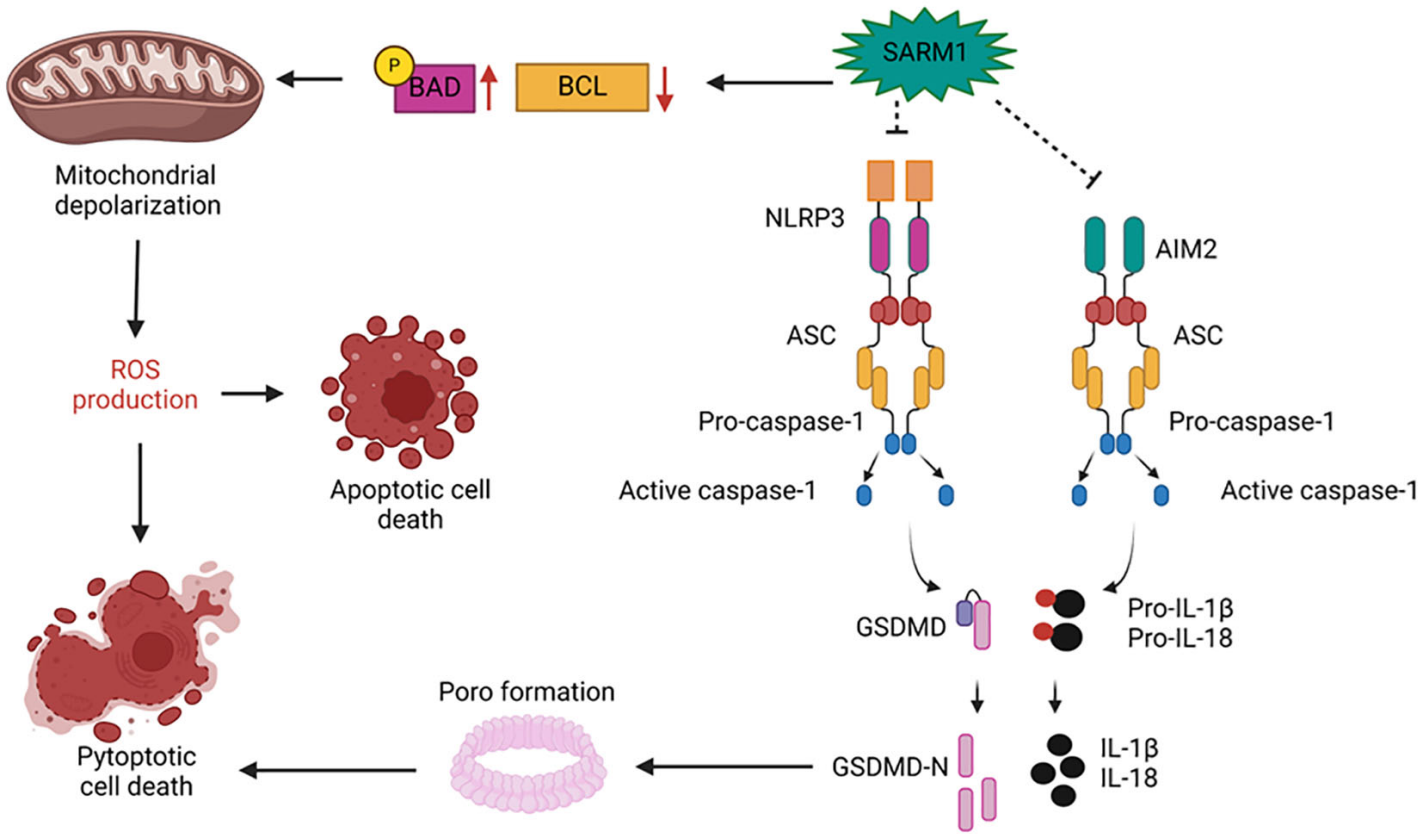
Schematic representation of SARM1 structure, its activation mechanism, and enzymatic activity. SARM1 consists of an autoinhibitory ARM domain, two tandem SAM domains responsible for SARM1 multimerization, a TIR domain with the NADase activity residue, and a mitochondrial membrane anchoring tag. The NMN/NAD+ ratio determines SARM1 activation status. A decrease in the ratio favors NAD+ binding to the ARM domains of SARM1 multimers, maintaining separation between the TIR domains of SARM1 multimers and inhibiting NAD+ hydrolysis, a process that promotes axonal survival in neurodegenerative diseases. A high ratio enables NMN binding to the ARM domain, induces conformational changes in the multimer, and brings its TIR domains closer to creating the allosteric NAD+ cleavage site, a process associated with neuronal degeneration progression after injury. **(C)** The catalytic activity residue of SARM1's TIR domain on NAD+ is located in the vicinity of amino acid 642. When activated, SARM1 degrades NAD+ into Nam and ADRP, the latter of which can still be cyclized to form cADRP. Figure created with BioRender.com.

SARM1 exerts negative regulation on NLRP3-mediated inflammation. Inflammasomes activation triggers the cleavage of GSDMD, Pro-IL-1B, and Pro-IL18, with GSDMD facilitating the formation of pores in cell membranes and enabling the release of matured cytokines from the platform (51–55). This can lead to highly inflammatory cell death, known as pyroptosis, which is an essential mechanism for eliminating replicative niches of intracellular pathogens and halting the inflammatory activity of the affected cell (56) (see **Figure 2**).

The regulatory impact of SARM1 on inflammasome-mediated inflammation is highlighted by its response to the intracellular pathogen *Klebsiella pneumoniae*, creating an anti-inflammatory environment conducive to bacterial proliferation. SARM1’s direct interaction with AIM2 impedes its activation, thereby disrupting the cleavage of Pro-IL-18, Gasdermin-D, and Pro-IL-1β in the presence of the pathogen. Deletion of SARM1 renders animals less susceptible to infection, underscoring its pivotal role in host defense. Furthermore, SARM1’s interaction with IRF3 and TRIF diminishes their activation, thereby attenuating the expression of inflammatory genes, including those encoding inflammasome components. Notably, as Klebsiella is a Gram-negative bacterium containing LPS in its cell wall, analogous outcomes have been observed in cells treated with purified LPS. Additionally, SARM1 promotes the production of IL-10, an anti-inflammatory cytokine, while concurrently reducing STAT3 phosphorylation, a key regulator of cytokine expression. This multifaceted modulation underscores SARM1’s intricate orchestration of the immune response (25).

Certain stimuli, such as LPG, a notable NLRP3 activator, induce robust inflammasome activation without triggering cell apoptosis. Despite this, the cell remains viable, producing elevated levels of pro-inflammatory cytokines. These cells enter a state of hyperactivation, which can sustain persistent inflammation. SARM1 plays a crucial role in preventing this phenomenon across various stimuli, including nigericin, where stimulated cells undergo programmed cell death, leading to transient inflammation. SARM1 achieves this by binding to NLRP3 via its TIR domain, thereby impeding the formation of ASC specks and reducing the availability of anchoring sites for caspase-1 activation. Furthermore, oligomerized SARM1, tethered to the mitochondrial membrane, undergoes augmentation following non-hyperactivating stimuli. This increase is associated with the induction of mitochondrial membrane depolarization and cellular energy collapse, signaling irreversible damage. Consequently, it triggers the shutdown of membrane repair mechanisms, facilitating the transition from the hyperactivated state to pyroptosis (26, 27) (see **Figure 2**).

In addition to promoting pyroptosis to prevent the generation of hyperactivated cells and directly binding to NLRP3 to decrease the activation of pro-inflammatory cytokines (26, 27), SARM1 plays a crucial role in inducing apoptosis in CD8 T cells, thereby contributing significantly to the contraction of their response. SARM1 exhibits heightened expression in naive T cells but significantly diminishes during activation. However, its expression is restored in terminally differentiated T cells, where it executes a pro-apoptotic function by reducing ERK phosphorylation, a pivotal determinant in T cell survival, proliferation, and differentiation. Additionally, SARM1 contributes to mitochondrial membrane depolarization and ROS generation. This process is facilitated by SARM1’s capability to downregulate the expression of BCL-xL, a crucial component in maintaining mitochondrial integrity and cell survival (28). Furthermore, SARM1 acts as a scaffold, promoting the recruitment of JNK3 to the mitochondrial membrane, a protein capable of inhibiting BCL2 and BCL-xL, phosphorylating and oligomerizing the pro-apoptotic protein BAD, and translocating the pro-apoptotic protein BAX to the mitochondria, thus reinforcing its role in promoting programmed cell death (57), a protein capable of binding and inhibiting BCL2 (58) and BCL-xL (59), promoting the phosphorylation and oligomerization of the pro-apoptotic protein BAD (60), and translocating the pro-apoptotic protein BAX to the mitochondria (61), supporting the role of SARM1 in promoting programmed cell death (See **Figure 2**). Overexpression of SARM1 leads to decreased ERK phosphorylation, resulting in increased ROS levels, reduced expression of BCL-xL, compromised mitochondrial integrity, decreased mitochondrial membrane potential, and ultimately, induction of cell death. Conversely, inhibition of ERK or overexpression of BCL-xL reverses this scenario, promoting cell survival. Inhibiting SARM1 expression via RNA interference enhances the survival of activated T cells and reduces Activation-induced cell death (AICD). Reduced expression of SARM1 is observed in certain types of lymphoma, correlating with the organism’s inability to effectively clear lymphocytes (28).

## 4 SARM1 NADase activity

**Figure 3:**
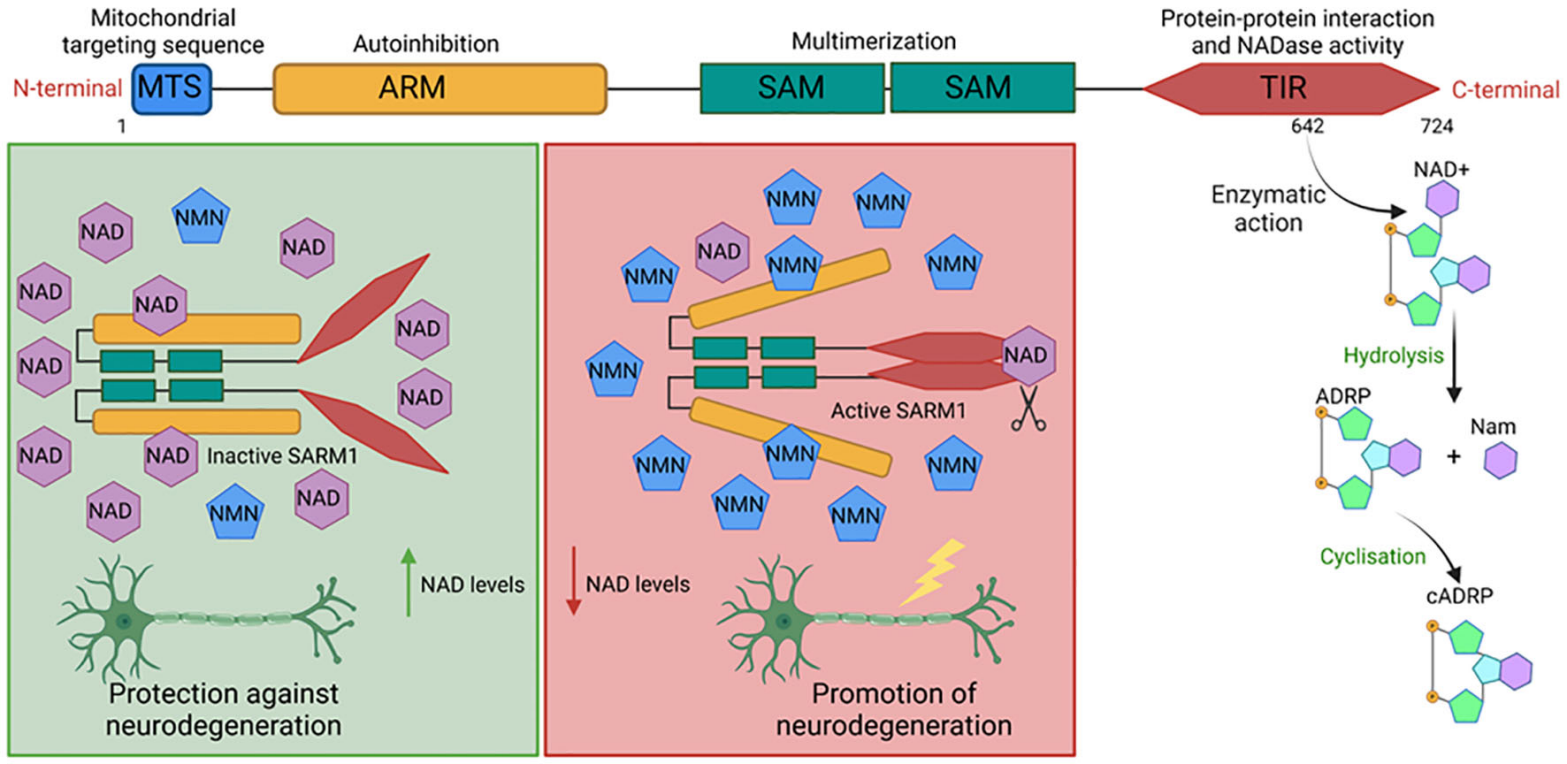
Control of different cell death pathways by SARM1. Upon its activation, NLRP3 and AIM2 inflammasomes recruit the adaptor protein ASC to form cleavage and activation platforms for caspase-1, a protease responsible for the maturation of pro-inflammatory cytokines IL-1β and IL-18, as well as for the cleavage of GSDMD. The N-terminal fragments of GSDMD form pores in the plasma membrane to promote the release of matured cytokines from the inflammasome and may lead the cell to pyroptosis. SARM1 can interact with NLRP3 and AIM2 molecules and prevent the formation of both inflammasomes. However, SARM1 can induce apoptosis or even pyroptosis. It is associated with the phosphorylation of pro-apoptotic proteins such as BAD and the reduction in the expression of proteins that maintain mitochondrial homeostasis, such as the BCL family proteins. This phenomenon induces increased ROS production to promote apoptotic cell death and, depending on the stimulus, to promote pyroptosis of cells to prevent the formation of hyperactive cells. Figure created with BioRender.com.

SARM1 comprises 724 amino acids divided into 4 domains, including a TIR domain, an ARM domain, and two tandem SAM domains, which respectively act in the protein’s effector actions, oligomerization, and auto inhibition (62, 63). SARM1 also features a signal peptide present in its N-terminal portion that directs it to the outer membrane of mitochondria (see **Figure 3**). Indeed, SARM1 co-localizes with mitochondria, although the anchoring status appears dispensable for triggering its effector functions (64). In humans, the structure of SARM1 forms an octameric arrangement, with each SAM domain adopting a characteristic bundle of five α helices (α1 to α5) separated by a 10-amino-acid linker. Although SAM domains typically form open polymeric structures, in SARM1, the rigid linkage results in a ring formation (65).

In the TIR domain of SARM1, a residue with NADase activity has been identified. Mutations that promote the substitution of amino acid 642 of this protein are sufficient to abolish the ability of SARM1 to cleave NAD+, indicating that its catalytic site is located in this region. This enzymatic activity is made possible by SARM1’s ability to form multimers, which leads to the approximation of SARM1’s TIR domains and the formation of the allosteric site that promotes NAD degradation. This was demonstrated by identifying that the ectopic expression of mammalian TIR domains incapable of dimerizing were deficient in performing this effector function (66). Activated SARM1 hydrolyzes NAD+ into Nicotinamide (Nam) and adenosine diphosphate ribose (ADPR) or the cyclic form of ADPR (cADPR), which serves as a biomarker of SARM1 activity. Other enzymes such as CD38, poly (ADP-ribose) polymerase (PARPs) play an important role in cellular NAD+ degradation (67, 68) (see **Figure 3C**).

In homeostatic conditions, SARM1 exists in an inactive state, forming octamers. At this stage, physical separation of TIR domains by auto-inhibitory ARM domains occurs, preventing the interaction of TIR domains essential for triggering its catalytic activity. This inactivation process is finely regulated by intracellular concentrations of NAD+ and nicotinamide mononucleotide (NMN). NAD+ serves as an inhibitor, whereas NMN acts as an activator of the complex. Both molecules compete for the allosteric pockets present in the ARM domains. Consequently, the component with a higher concentration is more likely to bind to this region, thereby controlling the activation status of SARM1 (69, 70) (see **Figure 3**). NAD+ is a cellular factor of extreme importance for metabolism and energy production, with its reduced form (NADH) serving as a critical intermediate for energy transfer between different metabolic pathways (71). Obtained through the transformation of nicotinamide (vitamin B3), NAD+ is an essential cofactor for other enzymes, such as ADP-ribose transferases, PARPs, and the sirtuin deacetylase protein (68, 72). NAD+ plays a crucial role in energy generation by electron transfer in glycolysis and the TCA cycle in the form of NADH (71).

The NADase activity of SARM1 is an important tool for promoting immunoregulation in innate immunity. PMA-differentiated macrophages derived from THP-1 cells, which express low levels of endogenous SARM1, produce high levels of IL-1β after stimulation with LPS and nigericin. The overexpression of wild-type SARM1 in these cells, using a viral vector, completely blocks cytokine production. When SARM1 with mutations that remove the NAD+ cleavage site is overexpressed, this blockade is partial, indicating that the NADase activity of SARM1 is important in regulating IL-1β production, but other SARM1-dependent regulatory mechanisms are also involved in the process (73). IL-1β production is also regulated by SARM1 in Bone Marrow-Derived Macrophages (BMDM), as Sarm1-/- BMDMs produce higher levels of the cytokine in response to LPS stimulation. These cells also exhibit increased glycolytic activity. This compilation of information reveals important discoveries regarding new mechanisms by which SARM1 regulates the expression of genes associated with immune response in innate immune cells and underscores the importance of investigating these and other mechanisms related to SARM1 biology in adaptive immune responses (74).

## 5 The impact of SARM1 on neurodegenerative diseases

Axonal degeneration is a primary cause of many neurological diseases, including peripheral neuropathy (75), traumatic brain injuries (76), glaucoma (77), optic nerve injury (78), and other degenerative disorders. This axonal degeneration is the result and is dependent on a peculiar type of programmed axonal cell death, similar to apoptosis, called “Wallerian-like” or Wallerian degeneration. It occurs at the axonal end after traumatic injury, stress, or cellular injury (79). In summary, this form of post-injury cell death is characterized by a reduction in the level of NAD+, an essential molecule for ATP generation, maintenance of redox balance, and axonal regeneration (80).

During aging, there is a reduction in NAD+ levels within the cells, with the reduction of NAD biosynthetic pathways such as nicotinamide phosphoribosyltransferase (NAMPT) being one of the possible causes, an effect observed in mice that underwent acute removal of NAMPT in hippocampal neurospheres (68, 81, 82). The reduction in NAD+ is associated with mitochondrial defects and age-related diseases, including neurodegenerative diseases, which are greatly influenced by the NADase activity of SARM1 (66, 67).

The SARM1 molecule is known for its significant role in the development of degenerative diseases, particularly neurodegenerative disorders, primarily due to its intrinsic activity of degrading NAD+, which is dependent on its TIR domain (80). This degradation capability is capable of promoting axonal degeneration (66). After a neuronal injury, there is a reduction in the expression of NMNAT2, an enzyme responsible for NAD biosynthesis, leading to an increase in the precursor NMN and subsequently, an elevation in the NMN/NAD+ ratio. NMN then translocates NAD+ from the allosteric site on SARM1, destabilizing the interactions of the autoinhibitory ARM domain and activating the TIR NADase activity (70, 83). This reduction in NAD+ is associated with axonal damage, and thus, the hydrolysis of NAD+ caused by SARM1 is shown to favor neurological degeneration and induce axonal destruction of neurons (84, 85) (see **Figure 3**).

SARM1 is also directly related to the development of Parkinson’s disease, a neurodegenerative disease characterized by the degradation of dopaminergic neurons in areas of the brain involved in movement control, mainly in the substantia nigra located in the telencephalon. The pathogenesis of the disease may involve different pathways and mechanisms, such as alpha-synuclein protein oligomerization, oxidative stress, calcium homeostasis, neuroinflammation, and axonal transport (86). In experimental models of Parkinson’s disease, SARM1-deficient animals showed less degeneration of the axons of dopaminergic neurons compared to wild-type animals. Furthermore, the absence of SARM1 allowed for the subsequent rescue of morphological, biochemical, and behavioral phenotypes of the animals, thus implicating the involvement of SARM1 in disease development (87).

Amyotrophic Lateral Sclerosis (ALS) is another neurodegenerative disease that affects the motor system, presenting characteristics such as frontotemporal dementia, and, like Parkinson’s disease, axonal degeneration mediated by the Wallerian degeneration process is one of the factors in disease development. In an experimental model of ALS, SARM1-deficient mice showed attenuated axonal degeneration, and the cell bodies of motor neurons were also significantly protected. Although the absence of SARM1 did not impact the behavioral deficits caused by the disease, the survival of these animals increased compared to wild-type animals (88). Allelic variants that maintain SARM1 constitutively active are associated with the development of ALS in humans and mice. Mice expressing *SARM1 V184G*, an allele frequently found in patients with ALS, exhibited severe motor impairment 4 days after induction of expression via viral vectors (89). The expression of another gain-of-function variant of *SARM1*, *GoF Δ229-235*, observed in patients with ALS, in mouse neurons exhibited reduced survival under stress conditions (90).

The aging process leads to the decline of physiological functions and is directly related to the risk of developing some neurodegenerative diseases, such as Parkinson’s disease, Alzheimer’s, and Amyotrophic Lateral Sclerosis. In a Drosophila model developed to study aging-related issues, it was observed that age can be an important factor in susceptibility to chronic exposure to rotenone, which is associated with locomotor problems and loss of dopaminergic neurons, characteristics observed in many neurodegenerative diseases. Chronic exposure to rotenone results in the rapid activation of dSARM (SARM1 in fruit flies), accompanied by increased inflammatory response and the formation of reactive oxygen species, processes that accelerate neurodegeneration. The activation of dSARM and subsequent locomotor deficits are reversed in the presence of anti-inflammatory agents (91).

SARM1 has been implicated in various neurodegenerative diseases not only due to its ability to promote axonal degeneration but also because of its correlation with neuroinflammation. Alzheimer’s disease is a complex and multifactorial neurodegenerative disorder that affects the brain, leading to progressive symptoms of cognitive decline, including memory loss, reasoning difficulties, and behavioral changes. Key factors contributing to Alzheimer’s disease include the formation of beta-amyloid protein plaques in the brain, neuroinflammation, neurotransmission imbalance, genetic factors, and aging. In an experimental model of Alzheimer’s disease, mice with conditional deletion of Sarm1 in the central nervous system experienced delayed cognitive decline. Additionally, the deletion of SARM1 reduced the deposition of beta-amyloid protein and decreased TNFα signaling in the hippocampus of the animals, providing protection against the neuroinflammation characteristic of the disease (92).

Apart from neurodegenerative diseases, there are other types of neuropathies, such as chemotherapy-induced peripheral neuropathy (CIPN). CIPN is a leading cause of morbidity and the primary reason for dose reductions and discontinuations in cancer treatment. Preclinical evidence indicates that activation of the Wallerian degeneration pathway, driven by SARM1, is responsible for the characteristic axonopathy of this condition. In an experimental model of CIPN, mice deficient in Sarm1 had less axonal function loss compared to wild-type animals. Similarly, animals treated with irreversible pharmacological inhibitors of Sarm1 showed less damage from CIPN. Additionally, *in vitro* studies showed that Sarm1 inhibitors were able to protect human axons from chemotherapy-induced injuries (93). The loss of SARM1 inhibits axonal degeneration for weeks after the injury and improves cognitive outcomes in mice after traumatic brain injury and vincristine-induced peripheral neuropathy (66). Therefore, programmed cell death involved in axonal degeneration after mitochondrial dysfunction has been shown to be independent of apoptosis (94) but induced by NAD+ reduction via SARM1-TIR (66). For this reason, it has been termed by many authors as “Sarmoptosis” (94).

The mechanism by which SARM1 is capable of promoting axonal destruction occurs through the active hydrolysis of NAD+ (95). In models of traumatic injuries or injuries induced by vincristine in neurons, the Sarm1-TIR domain exhibited intrinsic NADase activity, cleaving NAD+ into ADP-ribose (ADPR), cyclic ADPR, and nicotinamide. The orchestrated NADase activity by Sarm1-TIR has been shown to be essential in axons to promote the depletion of axonal NAD+ and consequently axonal degeneration after injury. Therefore, Sarm1 is being seen as a promising therapeutic target in diseases affecting axons, such as PD and ALS (66).

In addition to promoting metabolic changes in the injured neuron, SARM1 activation may also have secondary effects that could exacerbate various diseases. Genetic variants of *Nmnat2* that impair the enzyme’s ability to produce NAD+ lead to robust and intermittent activation of neuronal SARM1, further intensifying its degeneration. This effect has been associated with an increased number of activated macrophages in the nerves of mice expressing these *Nmnat2* variants, which promote neuroinflammation and impact disease progression. Macrophage depletion protected the animals from the deleterious effects of these variants; however, no intrinsic role for SARM1 in macrophages was observed in this context (96). A similar phenomenon occurs in the skin, where neurons play a central role in immune tolerance and barrier maintenance. Neurons produce norepinephrine, which regulates T γδ cell-mediated inflammation and CD8+ T cell cytotoxicity. However, a strong inflammatory stimulus in the skin, such as the Toll-7 agonist, induces robust activation of T γδ cells and leads to neuronal loss in the skin, mediated by SARM1. Neuronal degeneration intensifies inflammation in the skin, creating a positive feedback loop that promotes both inflammation and neurogeneration. The pathology of psoriatic skin and the presence of IL-17A-producing T γδ cells were reduced in mice deficient in neuronal SARM1 (97).

These findings highlight distinct neuroimmune interactions mediated by SARM1 and emphasize the importance of understanding the mechanisms involved in axonal death and neuroinflammation in order to devise therapeutic strategies against neurodegenerative and inflammatory diseases. It is important to emphasize that SARM1 is a central element in this process, characterizing this molecule as a promising therapeutic target (93, 98, 99).

## 6 SARM1 as a therapeutic target to treatment of inflammatory and neurodegenerative disease

Promising therapeutic avenues for directly inhibiting the SARM1 molecule, including its NADase enzymatic activity, with the potential to mitigate neurodegenerative and anti-inflammatory processes, have been explored. These inhibitors represent a topic of great interest in clinical research as they may attenuate degeneration in various neurological conditions characterized by axonal injuries and subsequent cognitive decline (100).

A potent and selectively reversible SARM1 inhibitor derived from isoquinoline, named DSRM-3716, has shown inhibitory activity on SARM1. However, its efficacy is ensured only if administered within 3 hours after neuronal injury, restoring the Sarm1-/- phenotype. This restoration is marked by an increase in NAD+ availability, decreased cADPR (a biomarker of SARM1 NADase activity), as well as protection of axons already destined for degeneration and recovery of axons already in an intermediate stage of degeneration. However, in a 2022 study by the same group, a molecule called 1AD was identified as responsible for inhibiting the NADase enzymatic activity of SARM1 by DSRM-3716. This molecule is formed *in situ* from a base exchange of the Nam portion of NAD+ by the previously studied small compound DSRM-3716. Thus, this study was able to unravel the molecular mechanisms of SARM1 inhibition by the small molecule derived from isoquinoline and its molecular interaction (100).

The chemotherapeutic drug Paclitaxel, used for treating certain types of cancer, has adverse effects. It strongly induces the NADase activity of SARM1 in neurons, leading to neuronal degeneration, a condition known as chemotherapy-induced peripheral neuropathy (CIPN). CIPN is characterized by intra-axonal increase of Ca++ cation via cADPR, a result of NAD+ metabolization by SARM1 activity (101). In this context, cADPR acts as a signaling molecule capable of altering cytoplasmic Ca++ concentration by modulating intracellular calcium release through ryanodine receptor channels (RyRs) present in the Endoplasmic Reticulum and activating a non-specific cation channel permeable to calcium (TRPM2) located predominantly in the plasma membrane, increasing the intracellular concentration of this ion. However, the exact mechanism by which intra-axonal Ca++ increase promotes axonal degeneration is not fully understood yet. Therefore, antagonists or genetic and pharmacological inhibitors of the SARM1-cADPR pathway, such as 8-Br–cADPR (102), are a pharmacological therapeutic alternative with the potential to prevent Paclitaxel-induced axonal degeneration via SARM1.

SARM1 can also be modulated at the transcriptional level through the use of drugs such as Resveratrol. This drug can stimulate SARM1 expression in lung tissue of animals infected with Respiratory Syncytial Virus (RSV) and block the TLR-3/TRIF/IFNy axis responsible for the exacerbated response to the virus, which worsens the condition of infected patients (103). It was found that RSV infection decreases SARM1 expression and increases TRIF expression in a time-dependent manner, starting from 36 hours post-infection. However, in mice infected with RSV and treated with resveratrol, SARM1 became more expressed, whereas TRIF had reduced expression compared to untreated infected animals. The drug’s effect was lost in animals treated with siRNA, reinforcing that resveratrol acts by reducing inflammation caused by RSV through the modulation of SARM1 expression (104). Resveratrol also proved to be a potent inhibitor of the signaling pathway originating from TRIF, further emphasizing the importance of SARM1 in regulating the mammalian immune response, as well as its potential as a therapeutic target for inflammatory diseases (105).

## 7 Conclusion

**Figure 4:**
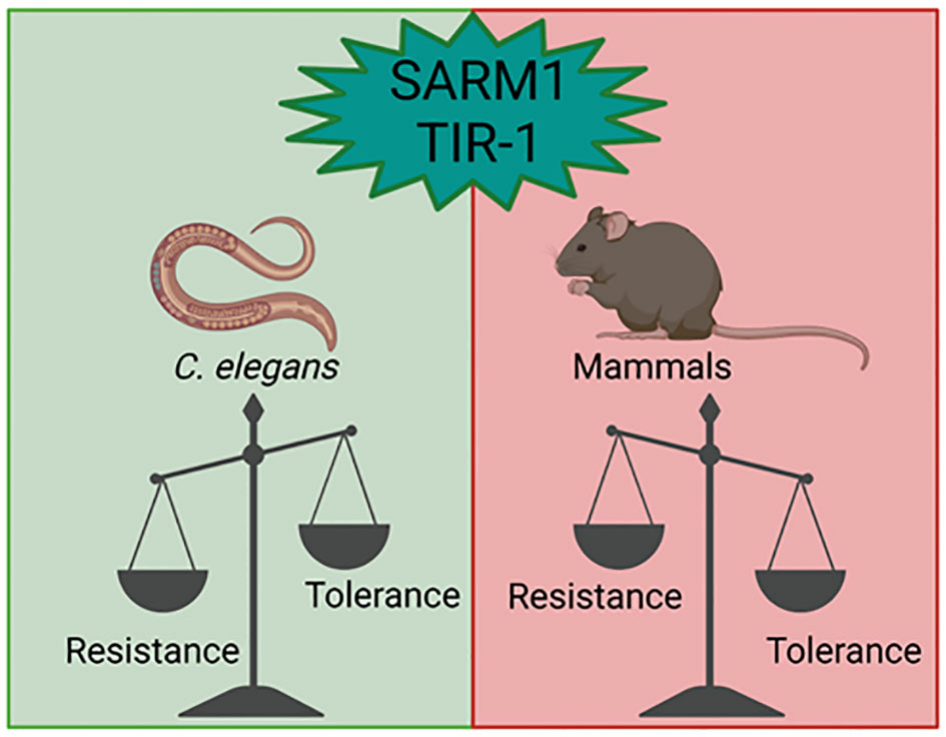
The importance of SARM1 in pathogen tolerance and resistance. SARM1 acts in different ways in invertebrates. In C. elegans, TIR-1 promotes inflammation and is essential for resistance to infections by microorganisms, while in human cells, SARM1 is associated with tolerance, aiming to prevent the exacerbation of the immune response and maintain the integrity of the host tissues. Figure created with BioRender.com.

In conclusion, the evolutionary conservation of SARM1 across phylogenetically distant organisms underscores its indispensable role in immune responses. Despite variations in effector functions, its structural preservation emphasizes its significance in maintaining body homeostasis. In invertebrates, it facilitates essential inflammation for host resistance, whereas in mammals, it regulates inflammatory responses and fosters tolerance (See **Figure 4**). While its NADase activity contributes to neurodegeneration, emerging evidence suggests its involvement in metabolic regulation within immune cells. Targeting SARM1 inhibition presents a promising therapeutic approach for immunopathologies, while its activation could enhance immune defenses against pathogens. Thus, understanding the multifaceted functions of SARM1 holds significant implications for advancing immunotherapy strategies and combating various diseases.
